# Determination of optimal experimental conditions for accurate 3D reconstruction of the magnetization vector via XMCD-PEEM

**DOI:** 10.1107/S1600577524001073

**Published:** 2024-02-19

**Authors:** Miguel A. Cascales-Sandoval, A. Hierro-Rodriguez, S. Ruiz-Gómez, L. Skoric, C. Donnelly, M. A. Niño, D. McGrouther, S. McVitie, S. Flewett, N. Jaouen, R. Belkhou, M. Foerster, A. Fernandez-Pacheco

**Affiliations:** a SUPA, School of Physics and Astronomy, University of Glasgow, Glasgow G12 8QQ, United Kingdom; b Institute of Applied Physics, TU Wien, Wiedner Hauptstraße 8–10, 1040 Vienna, Austria; c Departamento de Física, Universidad de Oviedo, 33007 Oviedo, Spain; d CINN, CSIC-Universidad de Oviedo, 33940 El Entrego, Spain; e Max Planck Institute for Chemical Physics of Solids, 01187 Dresden, Germany; f University of Cambridge, Cambridge CB3 0HE, United Kingdom; g ALBA Synchrotron Light Facility, 08290 Cerdanyola del Vallés, Spain; h Instituto de Física, Pontificia Universidad Católica de Valparaíso, Avenida Universidad 330, Valparaíso, Chile; i Synchrotron SOLEIL, L’Orme des Merisiers, 91192 Gif-Sur-Yvette Cedex, France; j Instituto de Nanociencia y Materiales de Aragón, CSIC-Universidad de Zaragoza, 50009 Zaragoza, Spain; Paul Scherrer Institut, Switzerland

**Keywords:** three-dimensional magnetic vector reconstruction, X-ray magnetic circular dichroism photo-emission electron microscopy, XMCD-PEEM, nanomagnetism, 360° domain wall rings

## Abstract

This work presents a detailed analysis of the performance of X-ray magnetic circular dichroism photoemission electron microscopy (XMCD-PEEM) as a tool for vector reconstruction of magnetization. For this, 360° domain wall ring structures which form in a synthetic antiferromagnet are chosen as the model to conduct the quantitative analysis. An assessment is made of how the quality of the results is affected depending on the number of projections that are involved in the reconstruction process, as well as their angular distribution. For this a self-consistent error metric is developed which allows an estimation of the optimum azimuthal rotation angular range and number of projections. This work thus proposes XMCD-PEEM as a powerful tool for vector imaging of complex 3D magnetic structures.

## Abstract 2

The performance of X-ray magnetic circular dichroism photoemission electron microscopy for vector imaging of complex three-dimensional magnetic textures is investigated.

## 1. Introduction

The field of nanomagnetism has evolved rapidly over the last few decades due to significant advances and developments in fabrication and synthesis methods (Fernández-Pacheco *et al.*, 2017 ▸). These improvements enable the fabrication of different magnetic systems with complex 3D configurations of the magnetization vector, as opposed to traditional simple mono-domain magnetic devices. The increase in complexity of magnetic systems (Vedmedenko *et al.*, 2020 ▸; Sander *et al.*, 2017 ▸) requires the adaptation and development of versatile characterization methods, where high magnetic sensitivity and spatial and temporal resolutions are some of the most important attributes.

Diverse laboratory-based characterization techniques are currently utilized to study the properties of materials via magnetic imaging, such as magnetic force microscopy (MFM) (Kazakova *et al.*, 2019 ▸), the different Lorentz transmission electron microscopy (L-TEM) modes (Phatak *et al.*, 2016 ▸; Fallon *et al.*, 2019 ▸), electron holography (Thomas *et al.*, 2008 ▸), scanning electron microscopy with polarization analysis (SEMPA) (Lucassen *et al.*, 2017 ▸; Unguris, 2001 ▸), spin-polarized low-energy electron microscopy (SPLEEM) (Rougemaille & Schmid, 2010 ▸; Suzuki *et al.*, 2010 ▸) and techniques which exploit the magneto-optical Kerr effect (MOKE) to perform wide-field (Flajšman *et al.*, 2016 ▸; Soldatov & Schäfer, 2017*a* ▸,*b* ▸) or scanning microscopy (Flajšman *et al.*, 2016 ▸).

Analogous to MOKE, although in the X-ray regime, synchrotron-based characterization techniques exploit the strong coupling that exists between photons and magnetism. X-rays offer high lateral resolution due to their short wavelengths, as well as element specificity that arises from the need to tune the photon energy to the absorption edge of the element in question. Imaging setups may be divided into two geometries, transmission and electron yield (Le Guyader *et al.*, 2012 ▸). Transmission X-ray microscopy (TXM) (Fischer *et al.*, 1998 ▸; Blanco-Roldán *et al.*, 2015 ▸), scanning transmission X-ray microscopy (STXM) (Zimmermann *et al.*, 2018 ▸) and coherent diffractive imaging (CDI) techniques such as ptychography (Shi *et al.*, 2016 ▸) and holography (Eisebitt *et al.*, 2004 ▸) all analyze the X-rays after passing through the magnetic mater­ial. Different strategies may be followed for tomographic reconstruction of the 3D magnetization vector (Donnelly & Scagnoli, 2020 ▸; Hierro-Rodriguez *et al.*, 2018 ▸; Donnelly *et al.*, 2017 ▸, 2018 ▸; Hierro-Rodríguez *et al.*, 2020 ▸), depending on the geometry and properties of the sample under investigation. This differs from photoemission electron microscopy (PEEM) or electron yield, where X-rays which have interacted with the material under investigation are not directly collected but rather the photoelectrons emitted as a consequence of such interaction. Due to the short electron mean free path, PEEM is an excellent candidate for investigating very thin structures close to the surface, *e.g.* the top layers of a multilayer heterostructure.

Previous work has utilized X-ray magnetic circular dichroism PEEM (XMCD-PEEM) to reconstruct the spatially resolved magnetization vector, by combining images taken at different relative X-ray/sample orientations (Le Guyader *et al.*, 2012 ▸; Ruiz-Gómez *et al.*, 2018 ▸; Ghidini *et al.*, 2022 ▸; Scholl *et al.*, 2002 ▸; Chopdekar *et al.*, 2013 ▸; Chmiel *et al.*, 2018 ▸; Digernes *et al.*, 2020 ▸). Here, we perform a detailed investigation of how the quality of the reconstructed 3D magnetization vector changes depending on the number of projections involved and their angular distribution. For this, 360° domain wall (DW) ring structures are chosen as the model to perform the reconstruction, given their small size which pushes the microscope’s resolution, and the complex winding sense of the magnetization. These textures are found to form in a synthetic antiferromagnet (SAF) multilayer heterostructure which shows interlayer Dzyaloshinskii–Moriya interactions (IL-DMI) (Fernández-Pacheco *et al.*, 2019 ▸). For further details of their formation the reader is referred to Sandoval *et al.* (2023 ▸).

In order to carry out this analysis, the algorithm first aligns the different projections with respect to each other, in such a way that they hold the same spatial orientation. A thorough analysis is then performed, which consists of running the reconstruction algorithm for different combinations of XMCD projections measured at different angles, applying to the resulting magnetization vectors an error metric that quantitatively gives account of the quality of the reconstruction. The resulting analysis allows optimization of the number of different rotation angles, as well as their specific orientation in relation to the desired accuracy of the magnetization vector reconstruction, being thus very useful for the design of time-efficient XMCD-PEEM experiments.

## 2. Methods

### 2.1. Experimental setup

The SAF layered structure investigated in this work consists of |Si/Ta (4 nm)/Pt (10 nm)/Co (1 nm)/Pt (0.5 nm)/Ru (1 nm)/Pt (0.5 nm)/CoFeB (2 nm)/Pt (2 nm)/Ta (4 nm)| (Fernández-Pacheco *et al.*, 2019 ▸), where the ferromagnetic layers are asymmetric in material and in thickness. The Co layer has dominating out-of-plane (OOP) anisotropy enhanced by the Pt layers at the interfaces, whereas the CoFeB layer’s thickness has been tuned slightly above its spin reorientation transition (SRT), showing moderately low in-plane (IP) anisotropy.

**Figure 1:**
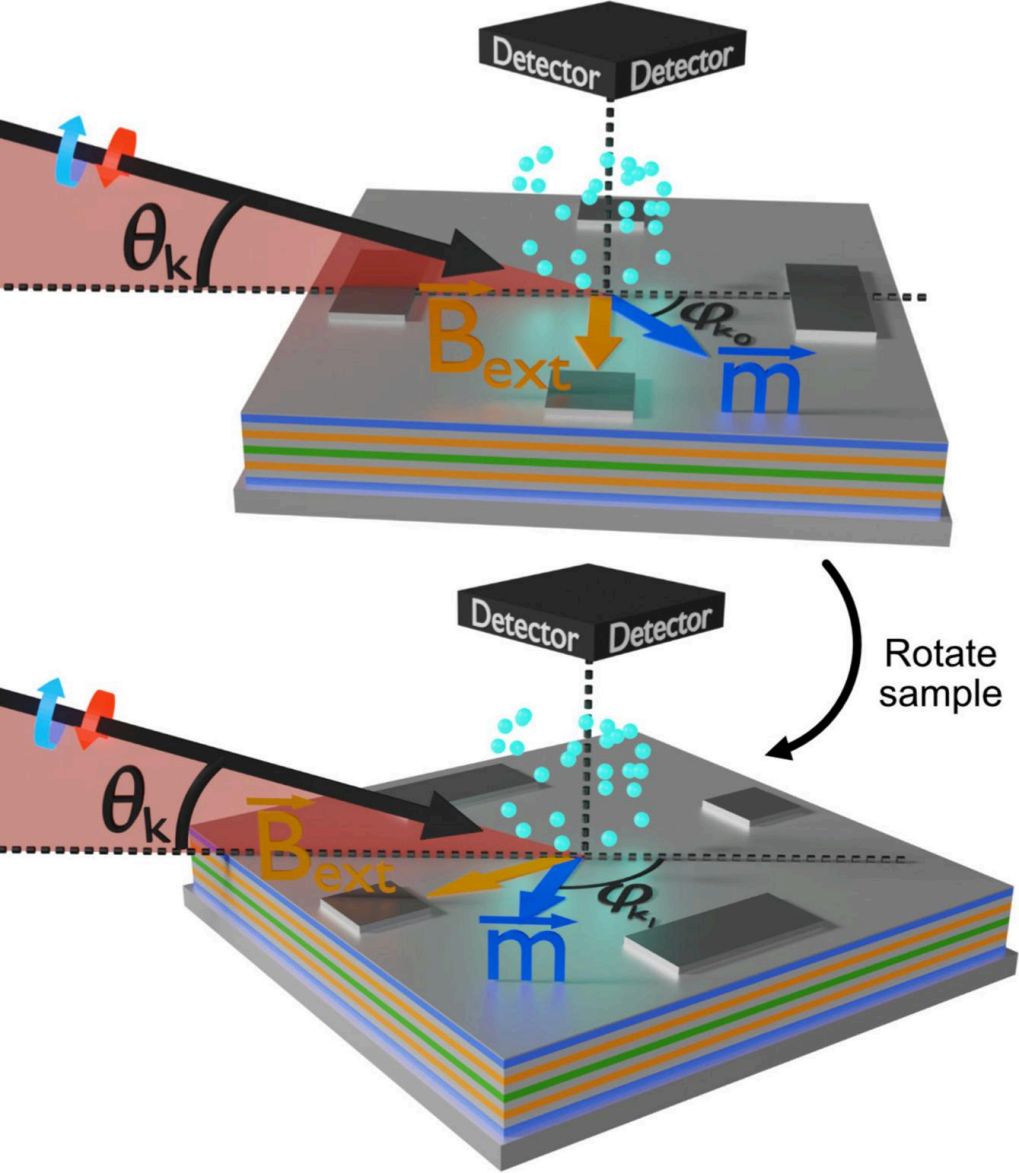
Diagrams describing the sample rotation with respect to the X-ray beam for measurement of different XMCD-PEEM projections. The X-ray wavevector **k** is given by the black arrow, the circular X-ray polarization eigenmodes by the blue and red circular arrows, the magnetization vector (**m**) by the dark-blue arrow and the external magnetic field (**B**_ext_) by the orange arrow. θ_*k*_ is the incidence angle with respect to the surface plane, and 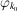 and 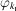 are the different relative angles between the X-ray beam and sample.

Prior to performing the synchrotron experiments, a series of repeating Pt_*x*_C_1–*x*_ patterns consisting of rectangles and squares were deposited via focused electron beam induced deposition (FEBID) on top of the film surface. Respectively, the sizes of the squares and rectangles are 1 µm × 1 µm and 2 µm × 1 µm, both being 50 nm thick. These are arranged in a square fashion, located at the midpoints of the sides of a 7 µm square as shown schematically in Fig. 1 ▸. They serve the purpose of providing a non-magnetic signal reference within the field of view (FOV), given that the magnetism-dependent photoelectrons do not possess sufficient energy to escape the sample’s surface through this additional bit of material. The non-magnetic signal reference is crucial for properly computing the final XMCD images, as there might be slight flux differences and flux spatial distribution when changing polarization, which would alter the number of emitted photoelectrons, thus inducing ficticious magnetic contrast. Hence, these corrections and references are crucial in order to be quantitative with PEEM.

The microscopy measurements were taken on the PEEM endstation of the CIRCE beamline at the ALBA Synchrotron (Aballe *et al.*, 2015 ▸). The sample is transferred to the PEEM chamber mounted on a holder with a dipolar electromagnet, providing the capability of applying IP uniaxial magnetic fields (Foerster *et al.*, 2016 ▸). It is mounted in such a way that the nominal easy axis (given by the Pt_*x*_C_1–*x*_ rectangle’s long axis) is aligned with the external magnetic field direction (**B**_ext_). The system allows rotation of the sample with respect to the surface normal, effectively changing the projection of the incoming X-ray beam onto the sample’s directions [Fig. 1 ▸]. Measuring at different X-ray/sample relative orientations provides sensitivity to different components of the magnetization vector, given that in XMCD-PEEM the magnetic contrast is proportional to **k** · **m** (Stöhr & Siegmann, 2006 ▸), with **k** and **m** representing, respectively, the X-ray wavevector and the magnetization vector.

### 2.2. XMCD image measurement and post-processing

The procedure followed in this work to obtain XMCD images is very similar to the one discussed by Le Guyader *et al.* (2012 ▸). After reaching the desired magnetic state, 256 images (acquisition time 2 s) are recorded for each incoming X-ray circular polarization in order to perform posterior averaging and improve the signal-to-noise ratio. Prior to the subsequent averaging of the same polarization images, a normalization is performed where each individual image is divided in a pixel-wise operation by a largely defocused image in order to remove channelplate contributions. The normalization image is obtained experimentally by going to a homogeneous area without obvious features, overfocusing the first lens (about 5%) and taking the average of 64 single images with the same settings as the real data. Once the channelplate contributions are removed, each polarization stack of images is individually aligned in order to correct for potential drifts during the time of measurement. For this, the Python *scikit-image* library (Van der Walt *et al.*, 2014 ▸) is used, where sub-pixel alignment is performed utilizing its Fourier-space cross-correlation algorithm. The alignment is done by selecting a region of interest (ROI) with a clear sharp feature, which in this case is chosen to be one of the FEBID deposited landmarks within the FOV. It is crucial to perform the channelplate correction prior to the alignment of each stack, otherwise artifacts due to the translation would be induced. In addition to the image alignment, within each polarization stack an equalization in image brightness is performed. For this, a proportionality factor that equalizes the average intensity in the Pt_*x*_C_1–*x*_ deposits for each image in the stack is found and applied as a global intensity factor to each full image. This is done to take into account and correct for potential X-ray flux variations during the time of measurement.

Averaging of the two aligned stacks of images is now performed, giving as a result two averaged images *I*_CL_ and *I*_CR_, corresponding to incoming circularly left and right polarization, respectively. The cross-correlation algorithm is utilized again for aligning these two images, and the intensity equalization is similarly done by finding a factor *f* which relates the intensity in the Pt_*x*_C_1–*x*_ deposits, *i.e. f* = 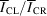 , with 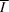 denoting the averaged intensity in the deposits. This factor accounts for changes in signal upon reversing the circular polarization. The final XMCD image is computed as *I*_XMCD_ = (*I*_CL_ − *f* *I*_CR_)/(*I*_CL_ + *f* *I*_CR_) (Stöhr & Siegmann, 2006 ▸), where these are all pixel-wise operations.

### 2.3. Magnetization vector reconstruction

To perform reconstruction of the three components of the magnetization vector, a minimum of three different projections are required in order to create a solvable system of equations with unique solutions. Experimentally, this is achieved by rotating the sample in the PEEM chamber about the sample normal and taking XMCD projections at different orientations, as sketched in Fig. 1 ▸. The XMCD images at each of the azimuthal angles are computed utilizing the procedure described in the previous section, although these host different spatial orientations due to the relative rotation between sample and camera. To correct for this, a new protocol which aligns the different azimuthal charge projections (computed as *I*_CL_ + *f* *I*_CR_) to one another is developed. Charge images are used for this, given that their contrast is independent of the magnetic configuration and azimuthal orientation, unlike the XMCD signals.

First, a single projection’s spatial orientation is chosen as a reference, with respect to which the rest of the projections are aligned. For this, the algorithm finds the most suitable affine transformation parameters: rotation, translation, scale and shear, which take the distorted projection to the reference. Scale and shear adjustments are necessary to correct image deformations introduced by the electron optics upon sample rotation. The error metric defined for this consists of the pixel-wise squared distance between both charge images, and the effectiveness of the procedure is further enhanced by applying a combination of Sobel edge and high-pass filtering algorithms to give more weight to the edges, which serve as alignment features. The optimized affine transformation parameters, which are found from running the algorithm on the charge images, are in the end applied to the corresponding XMCD images.

With the different projections now aligned, the magnetization vector is reconstructed by fitting at each pixel the associated XMCD azimuthal profile to the model, as given by expression (1): 

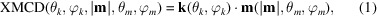

where θ_*k*_ and φ_*k*_ are the independent (or known) parameters which describe the normalized X-ray wavevector, corresponding to, respectively, the X-ray incidence angle with respect to the sample surface and the azimuthal rotation angle. These angles are known from the experimental setup. The remaining are the unknown (or fit) parameters: |**m**|, θ_*m*_ and φ_*m*_, which are the modulus, polar and azimuthal angles of the magnetization vector, respectively. Ten fits are done per pixel, where in each of these different random initial guesses are given to the fit parameters to avoid becoming pinned in local minima due to the parameter landscape.

### 2.4. Error metric and analysis

**Figure 2:**
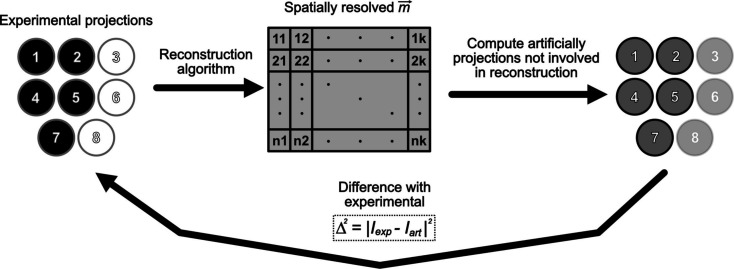
A schematic diagram describing the work flow of the error metric utilized for quantitatively assessing the quality of the reconstructed magnetization vector. A subset of the initial available experimental projections is taken; in this example three, six and eight are selected (left, white circles). The reconstruction algorithm is applied to obtain the spatially resolved vector given by the matrix, which is then utilized to compute artificially the projections that were not involved in the reconstruction (right, dark-gray circles). Finally, these artificially generated projections are subtracted in a pixel-wise operation from the experimental ones (black), squaring and summing for all the pixels, and normalizing by the number of images involved (in this particular case five). This error metric is represented by 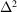 .

The main objective of this work is to investigate how the quality of the reconstructed results varies depending on the data used, *i.e.* not only the number of projections involved but also whether any particular combination of sample orientations is more beneficial than others. In order to be quantitative in this endeavor, an error metric needs to be defined. The procedure followed for this is sketched in Fig. 2 ▸, where eight is the total number of available projections (since this is the number measured experimentally). A combination of projections are picked, represented by white circles (with a minimum of three and a maximum of seven), which are then fed to the fitting algorithm to obtain a spatially resolved magnetization vector. With this vector configuration, the XMCD model is now applied in reverse, artificially generating the projections which were not involved in the reconstruction (black circles of the initial experimental projections). These artificially generated projections are now subtracted from their corresponding experimental real XMCD images. The resulting difference images are squared and summed, normalizing the resulting quantity by the number of images involved. The pixel-wise error metric corresponding to this process is mathematically described by 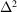 = |*I*_exp_ − *I*_art_|^2^.

An intuitive way of interpreting the meaning of this metric is the following. Utilizing part of the available experimental information, the reconstruction algorithm is run. Since the ground truth or real magnetic configuration is not known to compare how accurate the reconstruction is, the only comparison that can be made with real data is with respect to the other experimental projections. In order to do that, these are generated artificially utilizing the XMCD model, and compared in a pixel-wise operation.

## 3. Results and discussion

In previous work, ring-like structures were observed to form within the FOV of the SAF after applying IP demagnetization magnetic field procedures. Briefly, these protocols consist of applying oscillating fields of consecutively decreasing amplitude with a non-zero offset (Sandoval *et al.*, 2023 ▸). To perform vector reconstruction of the magnetization within these rings, eight projections were measured at the Co *L*_3_ edge (775.2 eV) with θ_*k*_ = 16° (large sensitivity to IP components). The signals obtained in this configuration are expected to come exclusively from the top CoFeB layer and not from the bottom Co, as the layered structure prevents the signal from the bottom Co layer from reaching the surface due to the short electron mean free path.

**Figure 3:**
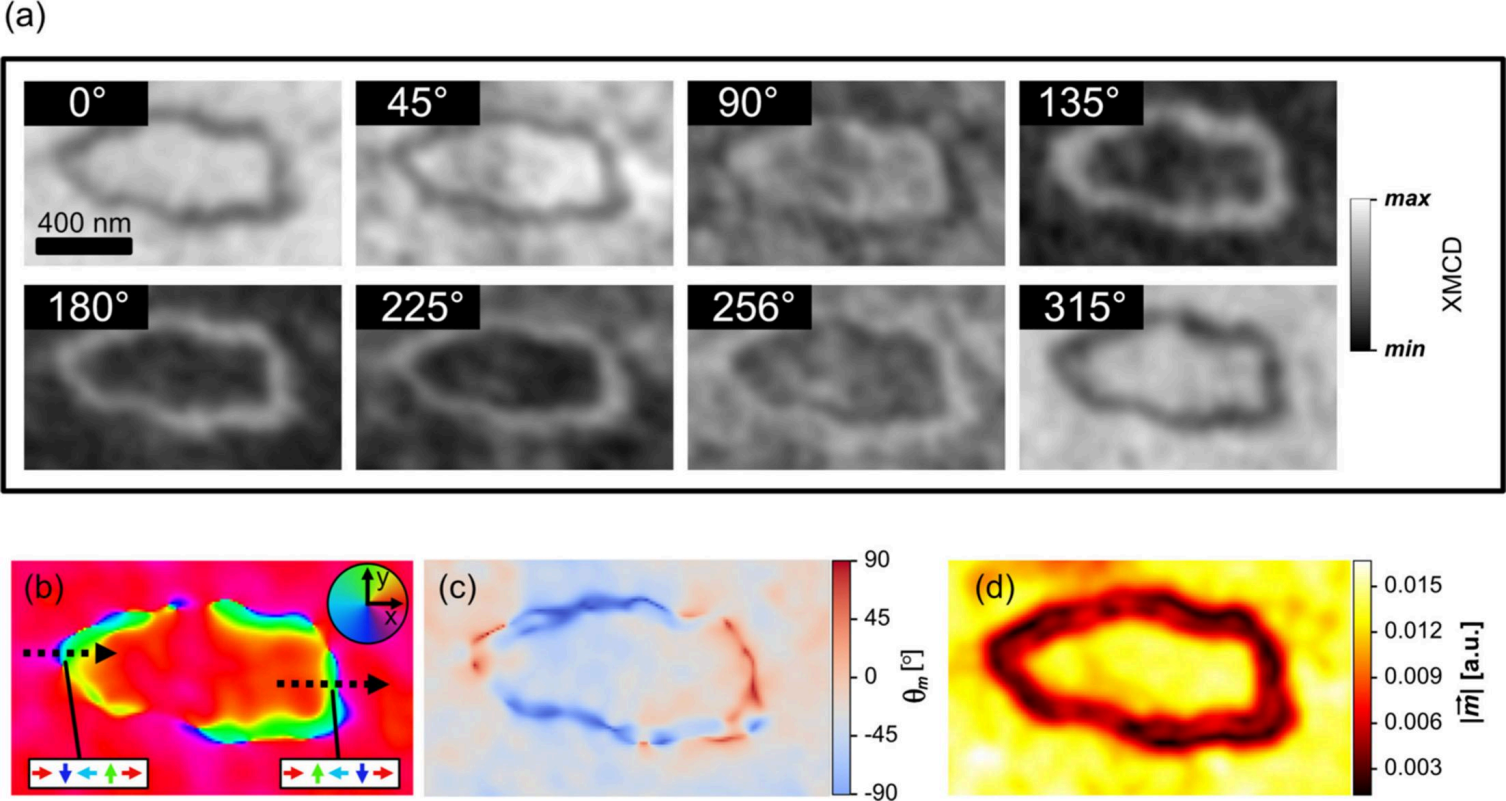
(*a*) Aligned experimental XMCD-PEEM projections, whose azimuthal rotation angles are given by the numbers in the inset. Images labelled 0° and 90° are parallel to the *x* and *y* directions of the inset in (*b*). (Bottom row) Spatially resolved (*b*) IP directions, (*c*) OOP component angle and (*d*) modulus of the reconstructed magnetization vector obtained from all eight experimental projections. The colored arrows in the white boxes of (*b*) denote the magnetization components along the dashed arrows.

The eight experimental projections are shown in Fig. 3 ▸(*a*), after having applied the image processing and projection alignment algorithms described in *Methods* [Sec sec2]. The magnetic signal in these images is determined as coming mostly from IP components, given that it varies upon azimuthal rotation (OOP magnetization would be insensitive to azimuthal rotation). The resulting 3D magnetization vector’s spherical components obtained after applying the reconstruction fitting algorithm to the eight projections are shown separately in Figs. 3 ▸(*b*)–3 ▸(*d*). The IP magnetization vector directions [Fig. 3 ▸(*b*)] reveal the presence of 360° DW rings separating the outer and inner domains, which point approximately along +*x*. The OOP component [Fig. 3 ▸(*c*)] is very close to zero in the uniformly magnetized areas, although it becomes significantly large in the DW area. A large uncertainty is expected for this component, mainly for two reasons. First, the very shallow angle of the incoming X-rays gives a small sensitivity to OOP magnetization (proportional to the sine of 16°). Second, on small length scales where the magnetization changes rapidly, the resulting magnetic signal measured by the microscope suffers a decrease in amplitude due to the microscope’s natural resolution (of the order of 30 nm; Aballe *et al.*, 2015 ▸). Thus even if, in reality, the signal is coming from IP magnetization, the decrease in amplitude makes the XMCD profile much more susceptible to noise deforming the expected sinusoidal form and preventing the algorithm from identifying it as such. The decrease in magnetic signal amplitude due to the microscope resolution is clearly evident in the spatially resolved modulus component [Fig. 3 ▸(*d*)], which becomes significantly smaller in the 360° DW (20–30% relative to the outer uniformly magnetized area). In the ideal case where the microscope had infinite resolution, the modulus of the magnetization vector would be constant throughout the probed space, given that it is made up of the same magnetic material (except if there were inhomogeneities and/or defects which could alter the saturation magnetization). Also, mis­alignment has a larger negative effect in the quality of the reconstructed results in areas where the magnetic features are of smaller length scales, *e.g.* in the ring.

**Figure 4:**
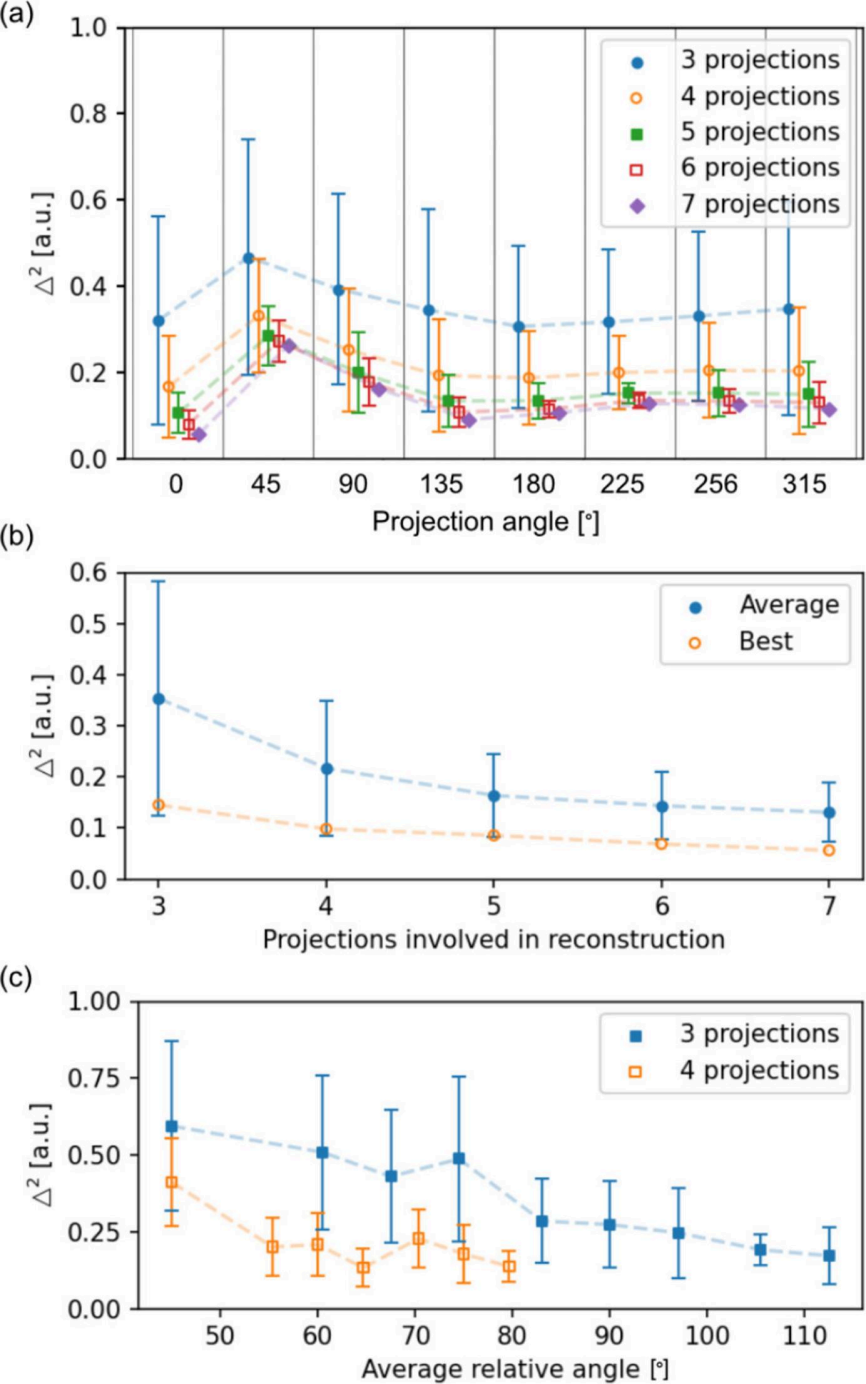
(*a*) A representation of 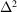 for the projections whose azimuthal angle is shown in the *x* axis of the plot, for different numbers of projections involved in the algorithm. (*b*) A representation of the average (filled circle) and lowest (empty circle) values of the error metric 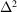 as a function of the number of projections involved in the reconstruction. (*c*) A representation of the 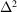 average with respect to the average relative angle in between projections involved in the reconstructions for three and four projections.

The previously described error metric, 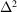 , is now computed and represented in Fig. 4 ▸(*a*) as a function of the projection azimuthal angle images displayed on the *x* axis. The points on the graph represent the average values of 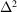 for all possible reconstruction combinations which exclude the projection at hand, while the error bars give the standard deviation or spread in 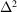 . This graph gives information regarding the quality of each individual XMCD projection, enabling identification of which of these are reliable, *i.e.* better levels of signal-to-noise ratio, smaller misalignments and deformations *etc.* Overall, the value of the error metric is of the same order of magnitude for all projections, which implies that the noise level and alignment between the different angles are quite similar, in all showing how the average error decreases as more projections are involved in the reconstruction. A particular case for these experiments concerns the case of the 45° projection, where the value of 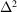 stands above all, having an even larger error for seven projections than in the rest of the azimuthal angles with three. This implies that the image quality at this angle in particular is worse than for the other angles, most probably due to imperfect correction and alignment with respect to the others. This error metric thus allows for detection of bad quality images which can be discarded from the final dataset if needed.

Now the error metric is plotted with respect to different relevant quantities in Figs. 4 ▸(*b*) and 4 ▸(*c*), as described hereafter. In Fig. 4 ▸(*b*), the curve of filled blue circles shows the average error for all the possible reconstruction combinations as a function of the number of projections involved in the algorithm. The curve of open orange circles represents the smallest error obtained for a single combination of projections, *i.e.* the best case for each projection number (shown in the supporting information). In both curves the error decreases as more projections are added to the reconstruction, due to two main factors: an increase in statistics, *i.e.* improving the signal-to-noise ratio, and from appropriately selecting the azimuthal angles of the projections involved in the reconstruction.

The best-case curve is mostly influenced by the increase in statistics, as the optimum azimuthal projection configuration has already been chosen. On the other hand, the blue curve’s errors are affected by both statistics and projection angles, as all the possible azimuthal combinations are considered. The effect of selecting the azimuthal projection angles on the quality of the reconstruction is depicted in Fig. 4 ▸(*c*), where 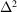 is represented against the average relative angle in between projections (considering three and four projections). A very clear trend is observed, which indicates how the error decreases as the spacing between projections becomes larger, converging to similar values for the largest separation possible. This is because the more spread out the projections are, the more evenly the different components are probed, having a lower average error in the vector field. Thus, these results reveal, as expected, that it is more effective to have fewer projections evenly spread in space than numerous projections spanning a narrow angular range. From the best case, five projections appears to be a good compromise between quality and time for measurements (each projection takes about 2 h of measurement including sample rotation). The error for five projections improves on the best-case error obtained with three projections by 42%, whereas for six and seven projections the improvements are by 52% and 61%, respectively.

## 4. Conclusions

In conclusion, we have quantitatively assessed how in XMCD-PEEM the quality of a reconstructed 3D magnetization vector depends on the number of projections involved and their spatial orientation. For this, we used 360° DW ring structures forming in a SAF multilayer as the model to perform a detailed analysis, measuring more than three or four projections, as is typically done in XMCD-PEEM. We have defined an error metric which uses part of the data for the vector reconstruction and the remainder for quantitative comparison.

As expected, the results show how the quality of the reconstructed vector improves significantly upon increasing the number of projections. More importantly, measuring these projections at azimuthal angles evenly spread through the full angular range improves the data quality more efficiently than pure statistics.

This quantitative approach provides the reader with an insight for the design of XMCD-PEEM magnetization vector reconstruction synchrotron experiments, where a compromise between accuracy in the reconstruction and the duration of the experiments becomes essential.

## Supplementary Material

Supplementary Information. DOI: 10.1107/S1600577524001073/gy5060sup1.pdf
