# Supplementary material for: Determination of optimal experimental conditions for accurate 3D reconstruction of the magnetization vector via XMCD-PEEM
Source: J Synchrotron Radiat. 2024 Feb 19;31(Pt 2):336–42. doi: 10.1107/S1600577524001073 (PMC10914169; doi:10.1107/S1600577524001073)
Supplement: Supplementary file 1 [file s-31-00336-sup1.pdf]

JOURNAL OF SYNCHROTRON RADIATION

Volume 31 (2024)

Supporting information for article:

Determination of optimal experimental conditions for accurate 3D reconstruction of the magnetization vector via XMCD-PEEM

Miguel A. Cascales-Sandoval, A. Hierro-Rodriguez, S. Ruiz-Gómez, L. Skoric, C. Donnelly, M. A. Niño, D. McGrouther, S. McVitie, S. Flewett, N. Jaouen, R. Belkhou, M. Foerster and A. Fernandez-Pacheco

SUPPLEMENTARY MATERIAL

Determination of optimal experimental conditions for accurate 3D reconstruction of the magnetization vector via XMCD-PEEM

Miguel A. Cascales Sandoval,[1,2] A. Hierro-Rodríguez,*[3,4] S. Ruiz-Gómez,*[5] L. Skoric,[6] C. Donnelly,[5] M. A. Niño,[7] D. McGrouther,[1] S. McVitie,[1] S. Flewett,[8] N. Jaouen,[9] R. Belkhou,[9] M. Foerster[7] and A. Fernández-Pacheco*[1,2,10]

*Corresponding authors e-mails: hierroaurelio@uniovi.es; Sandra.Gomez@cpfs.mpg.de; amalio.fernandez-pacheco@tuwien.ac.at.
[1]SUPA, School of Physics and Astronomy, University of Glasgow, Glasgow G12 8QQ, UK
[2]Institute of Applied Physics, TU Wien, Wiedner Hauptstraße 8-10, Vienna, 1040, Austria
[3]Departamento de Física, Universidad de Oviedo, 33007, Oviedo, Spain
[4]CINN (CSIC – Universidad de Oviedo), 33940, El Entrego, Spain
[5]Max Planck Institute for Chemical Physics of Solids, 01187 Dresden, Germany
[6]University of Cambridge, Cambridge CB3 0HE, UK
[7]ALBA Synchrotron Light Facility, 08290 Cerdanyola del Valles, Spain
[8]Instituto de Física, Pontificia Universidad Católica de Valparaíso, Avenida Universidad 330, Valparaíso, Chile
[9]SOLEIL Synchrotron, L'ormes des Merisiers, 91192 Gif-Sur-Yvette, Cedex, France
[10]Instituto de Nanociencia y Materiales de Aragón, CSIC-Universidad de Zaragoza, 50009 Zaragoza, Spain

1. Best reconstruction as a function of the number of projections

Fig. S1 shows the reconstruction with the lowest error as a function of the number of projections considered in this work. For each reconstruction, the spherical coordinates of the spatially resolved magnetization vector are shown separately, *i.e.*, modulus, in-plane (IP) and out-of plane (OOP) angles. Big changes are clearly observed when increasing from 3 to 4 projections in all three spherical components, specifically, the ring's surface is significantly increased and the OOP angle's sign is changed. Relative changes become smaller upon further increasing the number of projections, as the ring's surface continues to grow and finer magnetic texture is resolved evidenced in the OOP component, converging to the reconstruction shown in the main text with 8 projections.

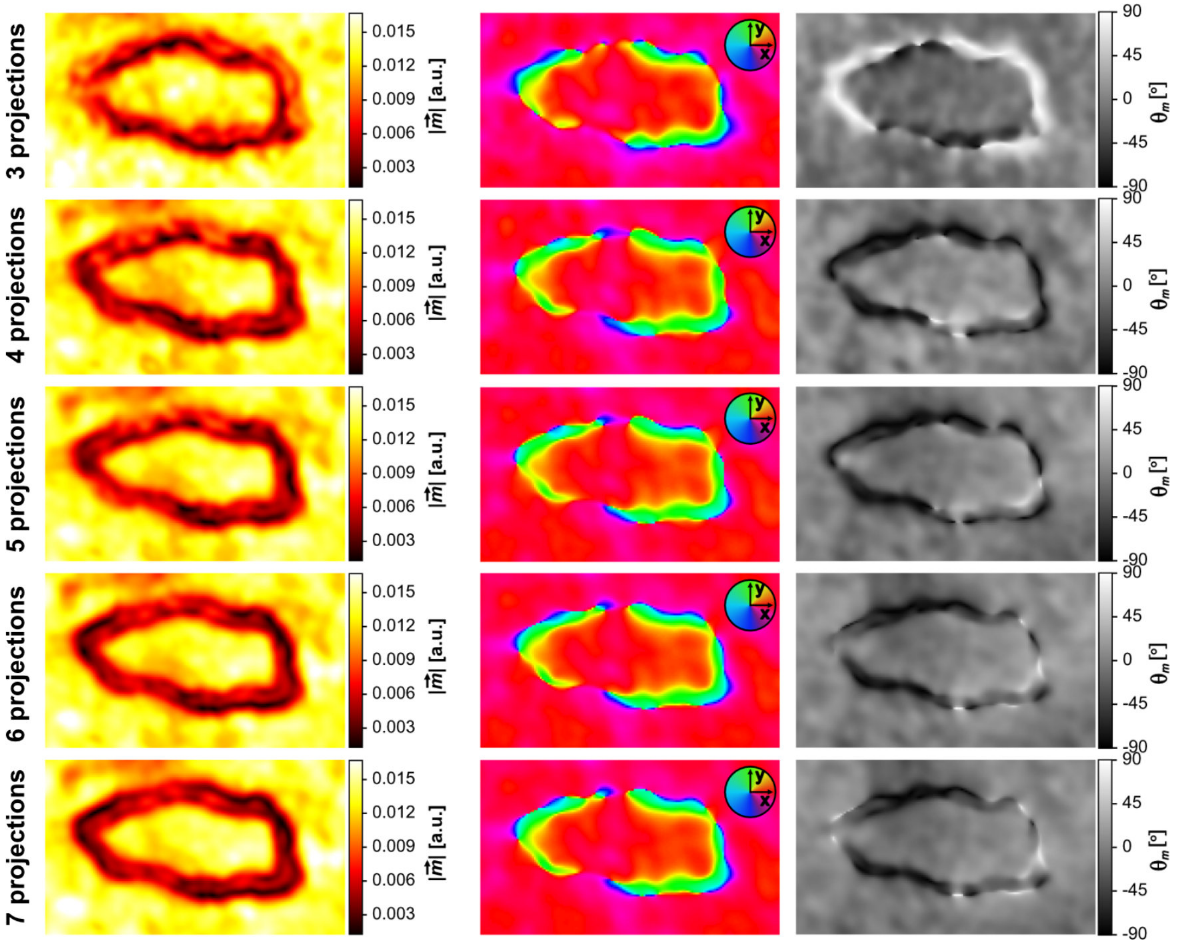

Fig. S1. Best reconstructions for different number of projections specified by the labels on the left. From left-to-right at each row: spatially resolved modulus, IP magnetization direction in *hsv* colormap and OOP component angle measured with respect to the plane of the film.
